# How social-emotional competence shapes classroom participation? A configurational analysis of teacher education students using fsQCA

**DOI:** 10.3389/fpsyg.2025.1695629

**Published:** 2026-01-05

**Authors:** Xiaofang Ma

**Affiliations:** Fuzhou University of International Studies and Trade, Fuzhou, China

**Keywords:** teacher education students, social–emotional competence, decomposing effect, classroom participation, fuzzy-set qualitative comparative analysis (fsQCA)

## Abstract

**Introduction:**

University students’ classroom participation is a research focus of great concern to educational scholars and university educators. Existing studies have indicated the potential impact of social–emotional competence on students’ in-class engagement, but the complex combinatorial effects of its components remain under-explored. This study aims to decompose the influence mechanism of university students’ social–emotional competence on classroom participation, specifically exploring how different combinations of social–emotional competence components jointly affect their classroom participation.

**Methods:**

A questionnaire survey was conducted among 172 teacher education students to collect data on their social–emotional competence and classroom participation. Fuzzy set qualitative comparative analysis (fsQCA) was adopted as the core research method to systematically explore the complex causal configurations between social–emotional competence components and high classroom participation.​

**Results:**

The analysis identified three distinct configurations of social–emotional competence that can effectively lead to high classroom participation among university students. Notably, self-awareness, relationship skills, and self-management emerged as the core conditional factors across all three configurations, playing irreplaceable roles in promoting classroom participation.

**Discussion:**

This study reveals the asymmetric and configurational nature of the relationship between social–emotional competence and classroom participation, enriching the theoretical understanding of non-linear influence mechanisms in educational psychology. The identification of core conditional factors provides practical implications for universities and educators to design targeted intervention strategies—such as strengthening self-awareness training and interpersonal skill development—to enhance students’ classroom participation. Future research could expand the sample scope across different disciplines and explore the moderating effects of teaching contexts on these configurations.

## 1 Introduction

In recent decades, social–emotional competence (SEC) has emerged as a cornerstone of holistic student development, with its significance extending far beyond classroom participation to shape broader academic success and well-being. This relevance is strongly anchored in key educational psychology theories that frame SEC as a foundational driver of adaptive learning behaviors. Self-Determination Theory (Ryan and Deci, 2000), for instance, posits that intrinsic motivation—critical for sustained academic engagement—depends on the satisfaction of psychological needs for autonomy, competence, and relatedness. SEC directly supports these needs: self-awareness enhances students’ ability to align learning goals with personal values (autonomy), self-management enables consistent effort toward challenging tasks (competence), and relationship skills foster positive peer and teacher interactions (relatedness). Empirically, studies have linked higher SEC to increased academic persistence (Zhang et al., 2022), better stress management during exams (Moradi and Chemelnezhad, 2021), and stronger peer collaboration in group projects (Hernández et al., 2016)—all of which contribute to improved academic performance.

Complementing this, the Broaden-and-Build Theory of Positive Emotions (Fredrickson, 2004) further illuminates SEC’s role in academic success by explaining how positive emotions—regulated and cultivated through SEC—expand cognitive flexibility and resource-building. For example, students with strong emotion-regulation skills (a core component of SEC) are less likely to experience performance anxiety during classroom discussions, allowing them to contribute more freely and retain information more effectively. Similarly, social awareness—another SEC dimension—helps students interpret peer and teacher feedback accurately, enabling targeted improvements in their learning strategies. Collectively, these theoretical frameworks and empirical findings establish SEC not merely as a “supplementary” skill but as a central mechanism that underpins both classroom participation and long-term academic achievement.

Past research has consistently demonstrated that classroom participation, as a key manifestation of SEC’s impact, yields multifaceted benefits for university students, spanning academic, cognitive, and long-term developmental domains. Academically, active participants in classroom discussions exhibit test scores up to 25% higher than their less engaged peers (Precourt and Gainor, 2019), while cognitively, participation fosters deeper content comprehension, critical thinking, and knowledge retention (Weaver and Qi, 2005). Beyond immediate learning outcomes, sustained classroom engagement predicts long-term success, including improved academic trajectories and career advancement (Ladd and Dinella, 2009; Finn and Zimmer, 2012), as it cultivates skills such as communication, collaboration, and problem-solving—competencies increasingly valued in modern workplaces. These findings collectively position classroom participation as a pivotal mediator between SEC and student success, underscoring the need to identify how SEC components interact to shape this engagement.

Despite the established link between SEC and academic outcomes, existing research on classroom participation has primarily focused on two sets of factors: contextual variables (e.g., class size, teacher assessment strategies, classroom climate) and surface-level individual traits (e.g., gender, shyness, fear of failure) (Fassinger, 1995; Crosthwaite et al., 2015; Mundelsee and Jurkowski, 2021). While contextual factors such as grading policies for participation can influence engagement (Rogers, 2011), researchers have noted that even when students recognize participation’s benefits for course grades, many remain reluctant to engage—suggesting that individual-level factors may exert a more proximal influence (Crosthwaite et al., 2015; Meyer, 2007). However, studies of individual factors have largely overlooked SEC, a malleable and multidimensional construct that is more amenable to intervention than fixed traits like shyness or introversion (Coplan and Rudasill, 2016).

A critical research gap thus emerges: few studies have examined how the components of SEC interact to shape classroom participation, and none have employed a methodological approach capable of capturing such complex, configurational effects. Most existing research on SEC and educational outcomes relies on regression analysis or correlation methods, which prioritize the independent, additive effects of single variables rather than exploring how combinations of SEC components may produce the same outcome through different pathways. This limitation is particularly problematic given that SEC is inherently multidimensional—its components do not operate in isolation, and their interplay is likely to drive participation in ways that single-variable analyses cannot capture.

To address this gap, the present study adopts fuzzy set Qualitative Comparative Analysis (fsQCA), a method uniquely suited to unpacking the configurational effects of multidimensional constructs. Unlike traditional regression or correlation analysis, which is constrained by assumptions of linearity, independence of variables, and sensitivity to multicollinearity, fsQCA offers three key advantages for studying SEC and classroom participation. First, fsQCA is a case-oriented, configurational method that focuses on combinations of conditions rather than individual variables. This aligns with the theoretical understanding of SEC as a system of interacting skills—for example, fsQCA can identify whether high participation arises from “self-management + social awareness + relationship skills” or “self-awareness alone (in the absence of other SEC components)”—revealing multiple, equifinal pathways to the same outcome.

Second, fsQCA is robust to small-to-medium sample sizes (Rihoux and Ragin, 2009), making it ideal for studies of individual-level student behavior (e.g., the 172-participant sample in this study). In contrast, regression analysis often requires large samples to detect small effect sizes and may produce unreliable results when variables are highly correlated (a common issue with multidimensional constructs like SEC). Third, fsQCA distinguishes between core and peripheral conditions, enabling researchers to identify which SEC components are “non-negotiable” for participation (core conditions) and which play a supporting role (peripheral conditions). For example, fsQCA can determine whether self-management is a necessary core condition for participation, or whether it can be substituted by strong relationship skills in certain configurations.

By applying fsQCA, this study addresses the following research question: How do configurations of university students’ social–emotional competence components affect their classroom participation? In doing so, it not only fills the gap in understanding SEC-participation dynamics but also provides actionable insights for educators seeking to design targeted SEC interventions to enhance student engagement.

## 2 Literature review

### 2.1 Influencing factors of classroom participation

Referring to the definition of participation, one of the most widely accepted models of classroom participation at the present time is the division of classroom participation into behavioral, affective, and cognitive participation (Fredricks et al., 2004). While, there are some limitations in this definition. Based on a series of studies and discussions, Fredricks et al. (2004) argue that classroom participation is multidimensional, inclusive, and malleable, and that it is the result of a combination of individual and context. Most researchers agree that classroom participation is a multi-dimensional construct (Mundelsee, 2021). Synthesizing previous research, the factors affecting student participation can be summarized as contextual factors, individual factors, and there are interactions and effects between contextual and personal and factors.

#### 2.1.1 Contextual factors

In terms of contextual factors, firstly, prior research has shown that class size has a significant impact on classroom participation, for example, Fassinger (1995) argued that class size is inversely related to student participation in the classroom. Second, teachers are also an important factor in influencing classroom participation, such as teachers’ assessment strategies for students’ classroom participation (Crosthwaite et al., 2015; Rogers, 2011; Krieger, 2003), as well as teachers’ gender (Kathleen and Czekanski, 2013). Third, it is also very important for teachers and students to work together to generate a positive classroom climate that is supportive of participation (Fassinger, 1995; Mak, 2011; Morell, 2007; Rocca, 2010). In addition to above, the characteristics of the learning task also influence students’ classroom participation (Newmann, 1991; Guthrie and Wigfield, 2000).

#### 2.1.2 Individual factors

According to self-determination theory (Ryan and Deci, 2000), in the process of self-determination, based on an understanding of the needs of the organism and a flexible interpretation of external events, people are free to choose their actions rather than being coerced or controlled. Self-determination usually involves controlling people’s circumstances or outcomes, but self-determinists may also choose to relinquish control. Whether or not students participate in the classroom is thus the result of their own choices, as Khojasteh et al. (2018) found, and it sometimes happens that students believe that they will not participate in classroom activities even when the teacher counts classroom participation in their overall grade. Therefore the individual factors of learners are more important influences compared to the contextual factors.

In terms of individual factors, previous inquiries have explored the effects of factors such as gender and age on classroom participation (Fritschner, 2000; Aziz et al., 2018; Dancer and Kamvounias, 2005). Another very extensive study found that willingness to communicate is an important factor in classroom participation, especially in classroom of a second foreign language (MacIntyre et al., 1998; Wonder, 2021). In addition to this, there are many studies that have explored the influence of learners’ personality psychological traits on classroom participation, such as shyness (Coplan and Rudasill, 2016; Mundelsee, 2021), introversion (Cetinkaya, 2005; McCroskey and Richmond, 1987; MacIntyre et al., 1999), fear of failure (Mundelsee, 2021; Appleton et al., 2006; Hsu, 2015), and others.

However, it should be clear that physiological factors such as gender and age are almost unchangeable, and personality psychological traits such as shyness, introversion, and fear of failure are more stable characteristics; in contrast, learners’ social–emotional competence is more malleable and is more likely to be changed by interventions. Therefore, this study focused on the effects of social–emotional competence on classroom participation among university students.

### 2.2 Social–emotional competence

In the past research, some words similar to social–emotional competence have been mentioned, such as emotional intelligence (Salovey and Mayer, 1990), social emotional education(SEE) (Cohen, 2001; Reeves and Le Mare, 2017), emotional competence(Garner, 2010; Saarni, 2000), social emotional learning(SEL) (Schonert-Reichl and Weissberg, 2015) and so on, these words are different in definition, connotation and emphasis. Different researchers have put forward different theoretical models for these concepts. At present, the influential theoretical models about social–emotional competence are as follows:

#### 2.2.1 Big five personality traits

The Big Five personality model is a framework outlining the five core dimensions of personality and is the most commonly accepted theory of personality today. This model includes five aspects: conscientiousness, openness to experience, neuroticism/emotional stability, extroversion and agreeableness (McCrae and John, 1992). These five dimensions represent a wide range of categories, aiming at capturing most personality differences, and are determined by analyzing and summarizing adjectives commonly used to describe people’s personalities and behavior (John et al., 2008).

#### 2.2.2 Emotional intelligence model

The Emotional Intelligence Model focuses on how individuals perceive, regulate, and think about emotions. The Model includes four domains: perceive emotions, using emotions to facilitate thoughts, understanding emotions, and managing emotions (Salovey and Mayer, 1990; Mayer et al., 2016). This model covers the processes from basic psychological process to advanced comprehensive psychological processes. In the Emotional Intelligence model, competencies that appear earlier are located on the left side of the branch, while competencies that develop later are located on the right side of the branch. People with high emotional intelligence are able to make faster progress and acquire more specific competencies (Salovey and Mayer, 1990).

#### 2.2.3 OECD framework for social and emotional skills

The OECD’s Social and Emotional Skills Framework is organized around the Big Five personality traits and focuses on the cognitive, social, and emotional skills needed to succeed in modern life and meet the challenges of the 21st century, and it encompasses the following domains: task performance, emotion regulation, collaboration, open-mindedness, interactions with others, and composite skills (Chernyshenko et al., 2018). The framework focuses on two age groups, primary (10 years old) and secondary (15 years old), to allow for comparisons at different stages of development (OECD, 2018).

#### 2.2.4 Framework for social and emotional learning (SEL)

The Framework is a comprehensive framework that examines educators, families, and communities working together to support social and emotional learning (SEL). This theoretical framework contains five domains: self-awareness, self-management, social awareness, relationship skills and responsible decision making (CASEL, 2023).

The five competencies encompass interpersonal, intrapersonal and cognitive skills. The framework is comprehensive in that it encompasses skills, attitudes, and knowledge. CASEL emphasizes developmental appropriateness, but there are no learning progressions or explicit developmental indicators provided in the model, nor are there specific benchmarks related to age or grade level. At the same time, the framework recognizes the need to consider students’ ethnic and cultural backgrounds, and based on this, this study is an application of the framework to the social–emotional aspects of Chinese university students, and is able to further test the appropriateness of this framework for students from different cultural backgrounds.

Specifically, this study selects the five core domains of the SEL framework as the measurement dimensions of social–emotional competence, mainly based on two key advantages of the framework. First, as a comprehensive framework, it goes beyond the single focus on individual skills and highlights the collaborative support of educators, families, and communities in promoting social–emotional learning—this aligns with the practical context of university students’ development, where their social–emotional growth is often shaped by interactions across academic, family, and social environments, making the framework more applicable to the research scenario. Second, unlike the OECD Framework for Social and Emotional Skills, which is explicitly restricted to two specific age groups (10-year-old primary school students and 15-year-old secondary school students), the SEL framework has no rigid age or grade limitations. This flexibility allows it to be effectively adapted to the research on Chinese university students, a group with distinct developmental characteristics and needs that differ from primary and secondary school students, thus avoiding the constraints of age-specific frameworks and ensuring the relevance of the measurement dimensions to the research subjects.

### 2.3 Social–emotional competence and classroom participation

The link between social–emotional competence (SEC) and classroom participation is not merely correlational but is theoretically grounded in two foundational frameworks of educational and positive psychology: Self-Determination Theory (SDT) (Ryan and Deci, 2000) and the Broaden-and-Build Theory of Positive Emotions (Fredrickson, 2004). These theories collectively explain how SEC, as a malleable set of intrapersonal and interpersonal skills, shapes students’ motivational orientations and emotional resources—two core drivers of whether and how students engage in classroom activities.

#### 2.3.1 Self-determination theory

SDT posits that intrinsic motivation—critical for sustained, voluntary engagement in learning activities—depends on the satisfaction of three universal psychological needs: autonomy (a sense of volition in one’s actions), competence (a perception of mastery over tasks), and relatedness (a feeling of connection to others). SEC directly functions as a “bridge” between these needs and classroom participation by equipping students with skills to recognize, regulate, and act on their psychological needs in academic settings.

Together, these mechanisms illustrate that SEC does not influence participation in isolation; instead, it integrates intrapersonal (self-awareness, self-management) and interpersonal (relationship skills, social awareness) skills to fulfill the psychological needs that underpin intrinsic motivation for classroom engagement.

#### 2.3.2 Broaden-and-build theory

Fredrickson’s (2004) Broaden-and-Build Theory expands on this link by highlighting how SEC shapes students’ emotional experiences—another key determinant of participation. The theory argues that positive emotions (e.g., confidence, curiosity) “broaden” cognitive flexibility and behavioral repertoires (e.g., willingness to speak up, contribute creatively), while negative emotions (e.g., anxiety, self-doubt) narrow focus to defensive behaviors (e.g., silence, withdrawal). SEC acts as a “emotional filter” that cultivates positive emotions and mitigates negative ones, directly influencing participation.

#### 2.3.3 Integrative perspective

When integrated, SDT and the Broaden-and-Build Theory reveal that SEC influences classroom participation through two complementary pathways: need satisfaction (intrapersonal and interpersonal) and emotional resource management. Importantly, these pathways are not mutually exclusive—they interact to shape engagement. For example, a student with strong self-awareness (satisfying autonomy needs) might feel more confident to contribute (a positive emotion, per Broaden-and-Build), which then strengthens their relationship with peers (satisfying relatedness needs) and further increases their participation over time.

### 2.4 Application of fuzzy-set qualitative comparative analysis (fsQCA) in the field of education

As a research method centered on configurational thinking, fuzzy-set qualitative comparative analysis (fsQCA) has demonstrated significant adaptability in various topics within the field of education, leveraging its unique advantage in unpacking complex interactive causal relationships among multiple factors. It serves as a powerful tool for exploring mechanisms where “combinations of multiple conditions drive a single outcome.” From the perspective of existing application practices, the value of fsQCA has been validated across diverse educational scenarios and research themes:

In the realm of educational technology application, Song et al. (2024) employed fsQCA based on the Unified Theory of Acceptance and Use of Technology to analyze data from 342 K-12 science teachers. Their findings revealed that none of the seven factors constituted a necessary condition for teachers’ intention to use virtual experiments; instead, three distinct combinations of conditions formed driving pathways, breaking the limitations of traditional single-factor analysis.

In student development research, Ma et al. (2024) applied fsQCA to explore factors influencing online learning engagement (OLE) among 516 K-9 students from three provinces in Central and Eastern China. They identified eight configurations leading to high OLE, with some pathways showing significant differences across grade levels and genders.

In higher education studies, fsQCA has been used to analyze the interactive mechanisms of multiple conditions underlying the development of digital-professional integration capabilities among vocational college students. It has also been utilized to uncover three configurational pathways leading to academic burnout among college students, confirming that no single factor alone causes burnout and emphasizing the need to focus on the synergistic effects of conditions such as academic stress and low academic self-efficacy (Chen and Chen, 2025).

Cross-disciplinary methodological innovations further corroborate the adaptability and expansion potential of fsQCA. In interdisciplinary research on education and employment, Cai et al. (2025a, 2025b, 2025c) combined fsQCA with SEM and ANN to successfully identify multiple driving combinations of university students’ willingness for flexible employment in the post-pandemic job market, demonstrating the method’s potential for synergy with other quantitative techniques. Additionally, studies in non-educational fields have used fsQCA-ANN integrated methods to analyze nonlinear causal relationships, and their core logic aligns closely with the trend of “integrating advanced methods to capture complex relationships” in education (Cai et al., 2025a, 2025b, 2025c; Di et al., 2025). Even educational studies that do not explicitly adopt fsQCA—such as research on university students’ environmental conservation intentions using SEM-ANN—still underscore the need for diverse methods in studying complex relationships (Cai et al., 2025a, 2025b, 2025c).

Existing research collectively indicates that fsQCA applications in education have covered core topics such as “teachers’ behavioral intentions,” “students’ learning status,” and “development of educational competencies.” Its analytical logic—which downplays the independent effects of single factors and highlights the value of condition combinations—aligns perfectly with the research needs of multidimensional constructs like social–emotional competence (SEC). Just as the cultivation of SEC inherently involves collaborative efforts from multiple stakeholders, its impact on classroom participation will inevitably exhibit multidimensional interactive characteristics. However, gaps remain in current fsQCA applications in education: no existing studies have applied it to analyze the relationship between SEC dimensions and classroom participation, particularly lacking configurational perspective explorations targeting university students.

Against this backdrop, the selection of fsQCA in this study is supported by clear rationale and innovative value. On one hand, this method can address the limitations of traditional quantitative approaches (e.g., regression analysis, structural equation modeling) in capturing the “combinatorial effects of SEC dimensions,” enabling accurate identification of multiple pathways to high classroom participation. On the other hand, applying fsQCA to the study of the “university students’ SEC-classroom participation” relationship expands the method’s application scenarios in the field of individual development in higher education. Simultaneously, it provides a novel configurational analytical perspective for uncovering the mechanisms of SEC, enriching the research paradigm on the relationship between social–emotional competence and classroom participation.

### 2.5 Research gap

Existing studies have conducted multidimensional explorations in the fields of classroom participation and SEC, laying a solid foundation for subsequent research. However, there remain two key limitations in the current literature. First, the exploration of individual driving factors for classroom participation is insufficient: most studies focus on relatively fixed physiological or personality traits such as gender and shyness, while neglecting SEC—a core individual factor characterized by multidimensionality and malleability. Particularly, there is a lack of research on how various dimensions of SEC interact to influence classroom participation. Second, methodological limitations exist: previous studies linking SEC to educational outcomes mostly rely on traditional quantitative methods such as regression analysis and structural equation modeling (SEM). These methods emphasize the independent effects of individual variables, making it difficult to capture the complex causal pathways of classroom participation under the “combined effect” of SEC dimensions and unable to reveal the configurational characteristics of “multiple equivalent paths to high participation.”

The aforementioned limitations collectively form the core research gap of this study: the academic community has not yet analyzed the impact of the interaction mechanism between SEC dimensions on university students’ classroom participation from a “configurational perspective,” nor has it applied methodologies suitable for such complex causal relationships. Therefore, it is necessary to introduce fuzzy-set qualitative comparative analysis (fsQCA) to systematically explore how the combination patterns of SEC dimensions drive high classroom participation, thereby filling the dual research gaps of “dimension interaction” and “methodological adaptation” in the “SEC-classroom participation” relationship.

## 3 Research model

The fsQCA approach, rooted in Boolean algebra and configurational logic (Ragin, 2009; Fiss, 2011), is uniquely suited to address the research question of this study, with three distinct advantages that align with the exploration of social–emotional competence (SEC) and classroom participation. First, unlike traditional linear methods (e.g., regression analysis) that emphasize independent variable effects, fsQCA focuses on combinations of conditions, enabling the identification of multiple equifinal pathways to high classroom participation—an essential feature given SEC’s multidimensional and interactive nature. Second, fsQCA is robust to small-to-medium sample sizes (Rihoux and Ragin, 2009), making it ideal for analyzing the 172-participant data while avoiding issues of multicollinearity common in SEC-related studies. Third, it distinguishes between core and peripheral conditions, allowing for precise clarification of which SEC dimensions (e.g., self-awareness, self-management) play non-negotiable roles versus supportive roles in driving participation—providing nuanced insights that single-variable analyses cannot capture.

**Figure 1:**
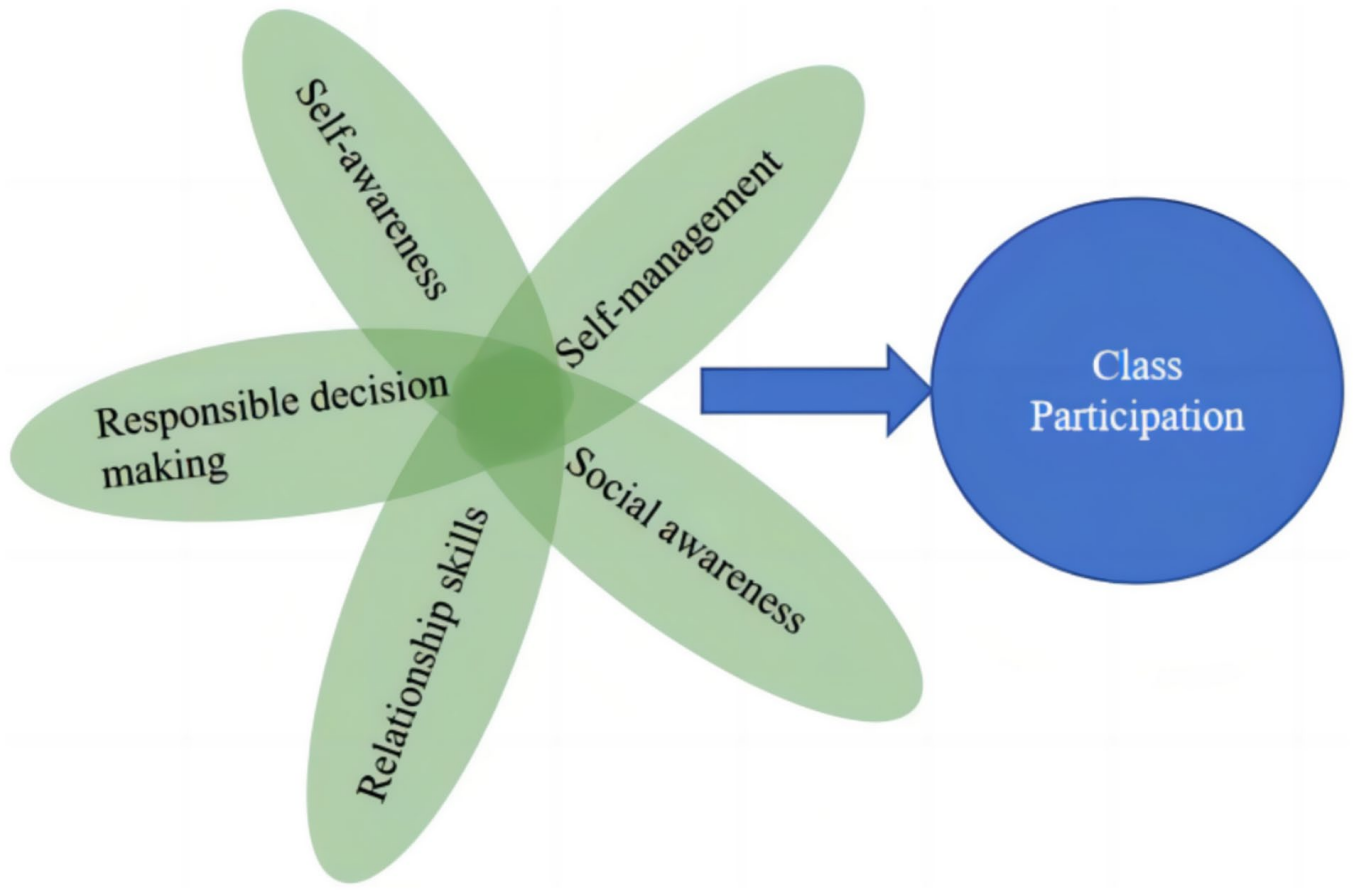
Conceptual model of class participation.

Based on the above literature review, this study proposes the proposition that social–emotional competence of undergraduate students on classroom participation and based on the content of the above literature review, social–emotional competence of undergraduate students is mainly measured through five variables: self-awareness (SA), self-management (SM), social awareness (SOA), relationship skills (RS), and responsible decision making (RDM). As a result, the following conceptual model (Figure 1) was formed as follows:


CP=f(SA,SM,SOA,RS,RDM)

## 4 Methodology

### 4.1 Data collection and respondents

The survey was conducted in July 2023 on “Question star”^1^, a Chinese website specializing in questionnaire surveys. As of August 3, 2023, the researcher received 172 completed questionnaires, all of which were tested to be valid. The study participants were all teacher education students, with the highest percentage of majors in elementary education and art education, including 36.83% in elementary education and 16.86% in art education. Due to the high percentage of female students in the teacher training program, there were only 17 male students (9.88%) among the study participants, and the rest were female.

After the questionnaire data was initially processed, descriptive statistics were derived as shown in Table 1.

**Table 1:** Descriptive statistical analysis of variables.

Variable	Mean	Standard deviation	Minimum	Maximum	Number of cases	Missing
Self-awareness (SA)	3.6488	0.5827	2.0000	5.0000	172	0
Self-management (SM)	3.4968	0.5700	1.5333	5.0000	172	0
Social awareness (SOA)	3.4994	0.5912	1.8000	5.0000	172	0
Relationship skills (RS)	3.4491	0.5822	1.9130	5.0000	172	0
Responsible decision making (RDM)	3.5099	0.6325	1.7143	5.0000	172	0

### 4.2 Measurements checking

The questionnaire for this study was divided into two parts: the first part was the social emotional competence questionnaire, which was adapted from the Emotional and Social Competency Inventory (ESCI) developed by McClelland Research and Innovation Center. The second part of the questionnaire is the teacher education student participation questionnaire, which was revised from the Student Classroom Engagement Questionnaire (SCEQ) (Handelsman et al., 2005). The questionnaire consisted of 94 items. In order to ensure the reliability and validity of the questionnaire, the study first conducted a reliability and validity test.

Firstly, the questionnaire was tested for reliability in the study and in the reliability test, the Cronbach’s *α* coefficient was 0.980 (Table 2), which is higher than 0.8, indicating that the questionnaire has good reliability. After that, the validity test of the questionnaire was conducted using KMO and Bartlett validity test, and the test results showed that the KMO value was 0.900 (Table 2), which was higher than 0.8, indicating that the questionnaire was very suitable for information extraction. In the Bartlett sphericity test, the *p*-value is 0.000 (Table 2), which is less than 0.05. This shows that the questionnaire has good validity.

**Table 2:** Results of reliability and validity tests of the questionnaire.

Reliability	Number of items	Number of cases	Cronbach’s α coefficient
	94	172	0.980
Validity	KMO values	0.900	
	Bartlett Sphericity Check	Approximate cardinality	15698.966
		df	4,371
		p -value	0.000

### 4.3 Fuzzy-set qualitative comparison analysis (fsQCA)

Fuzzy set qualitative comparative analysis (fsQCA) is a case-oriented research method based on the ideas of set theory and architecture, which effectively connects qualitative analysis with quantitative analysis. Its basic idea is to investigate the relationship between antecedent conditions and combinations of conditions and result variables from the perspective of set with the help of architecture theory and Boolean algebra operations, thus explaining the complex causal relationships behind the phenomena (Ragin, 2009; Fiss, 2011).

Compared with the traditional single case study, the advantages of fsQCA are mainly manifested in Rihoux and Ragin (2009): (1) fsQCA adopts multi-case study, and fully notices the complexity and heterogeneity of the individual cases themselves by establishing a pluralistic analytic composition of causal relationships. (2) Being able to analyze multiple causal combinations, identify subgroups of causal conditions that affect the emergence of a particular outcome, explore the equivalence between different subgroups of causal conditions, and separately analyze the subgroups of causal conditions in which a particular outcome occurs or does not occur. (3) Distinguishing itself from traditional qualitative research that explores the relationship of variables between cases, fsQCA is based on combinations of logistic conditions to compare different situations in the same model and between different models.

Compared with the traditional regression analysis oriented to independent variables and their effects, the advantages of fsQCA are shown in Rihoux and Ragin (2009): (1) fsQCA does not require high sample size and can systematically and effectively deal with multiple case comparative study data, which is more advantageous in small to medium sized samples where the variables consist mainly of dichotomous, definite and ordinal formulas. (2) fsQCA can analyze the role of combinations of causes on outcomes, which is not possible with regression analysis, where the independent variables are independent of each other and susceptible to the negative effects of autocorrelation and multicollinearity. (3) fsQCA emphasizes that there may be more than one combination of factors contributing to a given outcome, which in turn makes it possible to clarify the multiple ways and channels leading to that outcome and to measure the net impact of different combinations of causes on the outcome, allowing for more in-depth analyses of the causes contributing to the outcome in small to medium-sized sample analyses.

#### 4.3.1 Data calibration

In fsQCA analysis, the data of the independent and dependent variables need to be calibrated first, and the data need to be transformed into a fuzzy affiliation function between 0 and 1 by specifying anchors (Fiss, 2011). According to Ragin (2009) on anchor point setting, in this study the anchor point was set as (0.95, 0.5, 0.05) in this study. In other words, the upper quartile value was scored of 0.95 as the full membership, the median value was scored of 0.5 as an intersection, and the lower quartile value was scored of 0.05 as the full non-membership.

#### 4.3.2 Truth table establishment and refinement

*Establishing the truth table:* After calibrating the antecedent and outcome variables, the truth table was established. All possible logical combinations of conditions expressed as binary states of “present” or “absent” are listed according to the membership scores of the transformed fuzzy sets for all variables (Ragin, 2009).

*Editing the truth table:* The truth table has been edited by specifying the cutoff frequency and consistency thresholds. When the number of cases contained in the research is small, the frequency threshold should be 1 or 2, while when the number of cases is large, a higher frequency threshold should be selected (Ragin, 2009). In general, the cutoff value should not be less than 0.75, with a recommended cutoff value of ≥0.85 (Ragin, 2009). Therefore, in the study, the frequency threshold was set to 2 and the cutoff value was set to 0.85.

*Refining the truth table:* Later, the truth table was refined with the Quinn-McCluskey algorithm based on counterfactual analysis, which sets the effect of the antecedent variable on the outcome variable as “present or absent” (Fiss, 2011; Ragin, 2009). Three solutions to the problem (complex solution, parsimonious solution and intermediate solution) were obtained by this algorithm (Fiss, 2011; Ragin, 2009).

*Interpreting the solution*: Finally, by comparing the three solutions, core and peripheral factors were distinguished (Fiss, 2011; Ragin, 2009). This in turn explores the effect of the grouping of the different conditions on the outcome variable.

### 4.4 Complementary analysis

#### 4.4.1 Sensitivity analysis

Sensitivity analyses are used to check whether the results of the study are robust to conditions using alternative discriminant specifications (Fiss, 2011). In this study, sensitivity analysis was conducted by adjusting the anchor point system for data calibration by moving the anchor point up or down by 20%.

#### 4.4.2 Predict validity analysis

Predictive validity analysis is mainly used to validate the ability of the histogram model to predict the outcome variable under different data sets (Woodside, 2014; Olya and Altinay, 2016). In this study, the original sample was first divided by random selection into two approximately equal sub-samples: a modeling subsample (sub-sample 1) and a validation sub-sample (sub-sample 2). Then, fsQCA was performed on sub-sample 1 using the same number-of-cases threshold and consistency threshold as in the main analysis. Finally, the grouped models generated from sub-sample 1 were tested on sub-sample 2 to see if they achieved similar consistency and coverage as sub-sample 1.

#### 4.4.3 *Post hoc* analysis

Post hoc analysis is also considered as part of robustness testing, which refers to the introduction of the solution obtained from fsQCA into the framework of regression analysis through Tobit regression analysis, where the combination of relevant conditions obtained from fsQCA is transformed into the dependent variable, which can provide additional insights and complementary perspectives on the phenomenon under study. Thus, in this study, Tobit regression analyses were conducted separately for all combinations of results using classroom participation as the dependent variable and statements of social–emotional competence of university students as the independent variable.

## 5 Results

### 5.1 FsQCA results

The loudness factors for high classroom participation are shown in Tables 3, 4. The complex solutions, parsimonious solutions, and intermediate solutions for all configurations were shown in Table 3. In each configuration, the factors that appeared in the parsimonious solutions were the core conditions that led to the event, while the factors that appeared in the complex solutions but not in the parsimonious solutions were the peripheral conditions that led to the event (Fiss, 2011). As can be seen in Table 3, the core condition in the first configuration is Self-management (SM), the core condition in the second configuration is Relationship skills (RS), and the core condition in the third configuration is Self-awareness (SA).

**Table 3:** Results of the configuration analysis.

Configurations	Complex solutions	Intermediate solutions	Parsimonious solutions
Configurations 1	SOA*SM*SA*RS	SOA*SM*SA*RS	SM
Configurations 2	SOA*SM*RS*RDM	SOA*SM*RS*RDM	RS
Configurations 3	~SOA* ~ SM*SA* ~ RS* ~ RDM	~SOA* ~ SM*SA* ~ RS* ~ RDM	SA

“*” denotes Boolean logic “and”; “~” denotes Boolean logic “not”.

**Table 4:** Solutions for high classroom participation of university students.

Factor	Online teaching effectiveness		
	1	2	3
Self-awareness (SA)	●		●
Self-management (SM)	●	●	ⓧ
Social awareness (SOA)	●	●	ⓧ
Relationship skills (RS)	●	●	ⓧ
Responsible decision making (RDM)		●	ⓧ
Raw coverage	0.588896	0.63456	0.324766
Unique coverage	0.00324386	0.0577668	0.0674984
Consistency	0.867647	0.882373	0.903192
Solution coverage	0.714161		
Solution consistency	0.856502		

Note1: ● Means the “presence” of a condition and ⓧ means its “absence.” Large circles means core conditions. Small circles means peripheral conditions. Blank spaces mean that the condition is “indifferent” situation in which the causal condition may be either present or absent. Note2: Raw coverage: An fsQCA metric that quantifies the explanatory scope of a configuration, representing the proportion of cases with the target outcome (e.g., high classroom participation) accounted for by this specific configuration. Unique coverage: An fsQCA metric that measures the exclusive explanatory contribution of a configuration, referring to the proportion of target outcome cases explained only by this configuration (not by other identified configurations).

Table 4 demonstrated the results of the fuzzy set qualitative comparative analysis (fsQCA) of high classroom participation in this study. The researchers used black circles (●) to represent the presence condition, and a circle with a cross (ⓧ) circles to represent the absence condition (Ragin and Fiss, 2008). Large circles represent core conditions and small circles represent peripheral conditions. Blank spaces represent “indifferent” situations in which causal conditions may or may not exist.

As can be seen in Table 4, the three solutions show acceptable consistency (≥0.8). Regarding the core condition, Solution 1 demonstrated a successful hybrid configuration with Self-management (SM) as the core condition, with Self-awareness (SA), Social awareness (SOA), and Relationship skills (RS) as the peripheral conditions that can result in high classroom participation. While the presence or absence of Responsible decision making has no effect.

In Solution 2, Relationship skills (RS) is the core condition that, paired with the peripheral conditions of Self-management (SM), Social awareness (SOA), and Responsible decision making (RDM), can also result in high classroom participation. In the solution, the presence or absence of Self-awareness (SA) is irrelevant.

Solution 3 demonstrates a completely different scenario, with Self-awareness (SA) as the core condition, accompanied by the absence of Self-management (SM), Social awareness (SOA), Relationship skills (RS), and Responsible decision making (RDM) conditions, which can likewise lead to high classroom participation.

### 5.2 Complementary analysis results

#### 5.2.1 Sensitivity analysis result

The sensitivity analysis in this study was carried out by means of adjusting the anchor point system of data calibration by +/−20%, and the solution has changed slightly, but the explanation of the solution remains essentially the same.

#### 5.2.2 Predictive validity result

**Figure 2:**
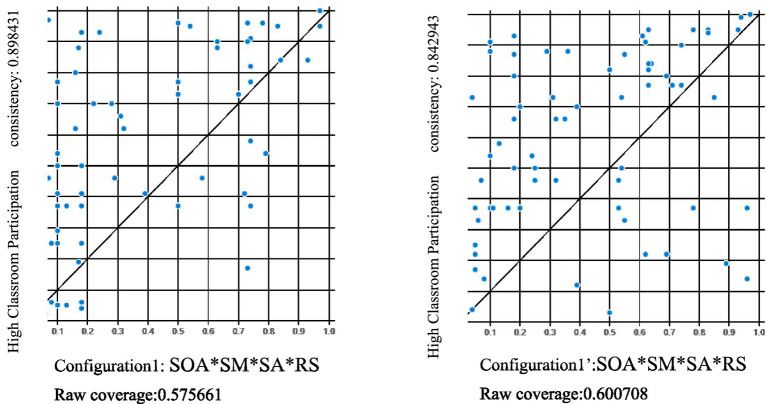
XY scatter-plots of university students classroom participation in configuration1 and 1′. Left Configuration 1 (by sub-sample 1); Right configuration 1′(by sub-sample 2).

In this study, the configurations in sub-sample 1 were examined using data from sub-sample 2. The results of the predictive validity analysis showed that the coverage and consistency of the results of all the model tests were very close (Figure 2 shows the model tests for Configuration 1). Thus, the proposed configurations university students high classroom participation were highly predictable across data sets.

#### 5.2.3 *Post hoc* analysis result

This study used Tobit regression analysis for *post hoc* analysis to examine the validity of the results. As shown in Table 5, the *p*-value for the second solution and the third solution is less than 0.05, indicating that Configuration 2 ( β =1.248693, P =0.0000), and Configuration 3 ( β =0.966101, P =0.0000) have an extremely significant effect on university students’ classroom participation. Whereas, the effect of Configuration 1 ( β = − 0.412951, P =0.0718) on university students’ classroom participation is insignificant. Since the principle behind fsQCA analysis is different from regression analysis, based on the above post hoc analysis results, the researcher believes that these results were correspond roughly as those derived from fsQCA.

**Table 5:** Results of the Tobit regression analysis for university students’ classroom participation.

Independent variable	Coefficient	Std. Error	z-Statistic	Prob.
Configuration 1	−0.412951	0.229356	−1.800482	0.0718
Configuration 2	1.248693	0.214554	5.819951	0.0000
Configuration 3	0.966101	0.106541	9.067918	0.0000

*P< 0.1;**P<0.05; ***P<0.01. (Fisher, 1925).

## 6 Discussion

### 6.1 Key findings

From the perspective of SEC components, this study identifies three configurational paths that drive university students’ high classroom participation. Below is an in-depth analysis of these paths, including their alignment with prior literature and theoretical frameworks reviewed earlier, as well as their unique contributions to expanding existing knowledge.

#### 6.1.1 Solution 1: self-management (SM) as the core, with peripheral support from SA, SOA, and RS

Solution 1 (SOA*SM*SA*RS) positions self-management (SM) as the core condition, while self-awareness (SA), social awareness (SOA), and relationship skills (RS) serve as peripheral conditions; responsible decision-making (RDM) has no significant impact on the outcome. This finding exhibits strong consistency with both theoretical frameworks and empirical studies highlighted in the literature review.

Theoretically, this path echoes the core logic of Self-Determination Theory (SDT) (Ryan and Deci, 2000). SDT posits that “competence” is a foundational driver of intrinsic motivation for academic engagement. Self-management, as defined by the SEL framework (CASEL, 2023), directly supports the satisfaction of this need: it enables students to maintain consistent effort amid academic challenges (e.g., staying focused during long discussions, regulating distractions to contribute thoughtfully). The peripheral role of SA, SOA, and RS further aligns with SDT: SA helps students align participation with personal learning goals (reinforcing autonomy), while SOA and RS facilitate positive interactions with peers/teachers (supporting relatedness)—but these factors only amplify participation when paired with the core “competence” support of SM.

Empirically, this path is consistent with prior studies linking SM to sustained academic engagement. For example, Moradi and Chemelnezhad (2021) found that strong SM skills help students manage exam stress and maintain learning motivation, while Hernández et al. (2016) noted that SM enhances peer collaboration effectiveness. Additionally, Song et al. (2024) identified “effort expectancy” (a construct analogous to SM) as a core driver of teachers’ technology adoption—reinforce that self-regulatory skills often function as the “linchpin” of behavioral engagement, with other social/cognitive skills playing auxiliary roles.

Notably, the irrelevance of RDM in this path aligns with the SEL framework’s flexibility. CASEL (2023) emphasizes that SEC dimensions do not operate uniformly across contexts: in scenarios where participation relies on consistent effort (rather than complex ethical or situational judgments), RDM becomes less critical—a nuance rarely highlighted in prior SEC-participation research.

#### 6.1.2 Solution 2: relationship skills (RS) as the core, with peripheral support from SM, SOA, and RDM

Solution 2 (SOA*SM*RS*RDM) identifies relationship skills (RS) as the core condition, with SM, SOA, and RDM as peripherals; SA is irrelevant here. This finding aligns with both the Broaden-and-Build Theory (Fredrickson, 2004) and empirical studies on social interaction’s role in participation, while also refining understanding of SEC’s context-dependent relevance.

Theoretically, Fredrickson’s (2004) framework explains why RS acts as a core driver: positive social interactions (facilitated by RS) cultivate positive emotions (e.g., belonging, confidence), which “broaden” cognitive flexibility and behavioral repertoires—encouraging students to speak up, contribute to discussions, and engage with peers. SOA (the ability to interpret others’ emotions/needs) and SM (regulating emotional responses to social feedback) reinforce this effect: SOA helps students adapt their contributions to group dynamics, while SM prevents anxiety from discouraging participation. RDM, though peripheral, supports participation by helping students make context-appropriate choices (e.g., when to listen vs. speak in collaborative tasks)—consistent with the OECD Social and Emotional Skills Framework’s emphasis on “interactions with others” as a key domain (Chernyshenko et al., 2018).

Empirically, this path validates prior research on RS and classroom engagement. Weaver and Qi (2005; Section 1) found that students with strong interpersonal skills are 30% more likely to participate in class discussions, as they feel more confident in social contexts. Similarly, Mundelsee and Jurkowski (2021) noted that collaboration-focused activities boost participation primarily for students with strong RS—echoing this path’s focus on RS as the core enabler.

The irrelevance of SA in this path is a notable extension of existing literature. Prior studies (e.g., Saarni, 2000) often frame SA as a “foundational” SEC dimension, but this path reveals that in socially oriented participation (e.g., group discussions, peer feedback), external social skills (RS, SOA) can compensate for weak internal self-awareness. This aligns with Zhang et al.’s (2022) observation that SEC dimensions exhibit “substitutability”—a concept rarely explored in configurational terms before.

#### 6.1.3 Solution 3: self-awareness (SA) as the core, with all other SEC dimensions absent

Solution 3 (~SOA* ~ SM*SA* ~ RS* ~ RDM)—where SA alone drives high participation, with all other SEC dimensions absent—is the most distinctive finding of this study. While it appears counterintuitive at first glance, it aligns with the theoretical essence of SA and the unique characteristics of teacher education students, while also addressing a gap in prior configurational research.

##### 6.1.3.1 Alignment with theoretical frameworks

This path deepens the application of SDT’s autonomy principle. SA, defined as the ability to recognize one’s emotions, strengths, and learning needs (CASEL, 2023), enables students to satisfy the need for autonomy independently of external conditions:

Students with strong SA can identify personal learning gaps (e.g., “I need to clarify my understanding of lesson planning”) and frame participation as a means to achieve self-directed goals (e.g., “Speaking up will help me practice teaching communication skills”). This intrinsic motivation reduces reliance on external supports like peer approval (RS) or stress regulation (SM).

Unlike RS (which depends on others’ responses) or SM (which reacts to external challenges), SA operates through “internal validation”: students with high SA judge participation based on personal growth (e.g., “Did this contribution advance my professional development?”) rather than external feedback. This aligns with Fredrickson’s (2004) note that positive emotions rooted in self-efficacy (a byproduct of SA) can independently drive engagement.

##### 6.1.3.2 Consistency with sample characteristics and prior group-specific research

The uniqueness of this path is also tied to the study’s sample—teacher education students. As prospective educators, this group undergoes systematic training in “reflective practice” (e.g., analyzing teaching methods, evaluating personal strengths/weaknesses), which amplifies SA’s predictive power.

Ma et al. (2024), who studied K-9 students’ online engagement, found that SA plays a more prominent role in groups with strong “goal orientation”—a trait common in teacher education students (who often link classroom activities to future teaching careers).

Chen and Chen (2025) who explored college students’ academic burnout via fsQCA, noted that SA can mitigate burnout even when other SEC skills are weak—suggesting SA’s potential as a standalone protective factor. This study extends this insight to participation, showing SA can also drive engagement independently.

##### 6.1.3.3 Distinction from prior configurational studies

Prior fsQCA research in education has focused on “synergistic configurations” of multiple factors, but none have identified a single SEC dimension as a sufficient core condition. This solution fills this gap by demonstrating that SA—when amplified by professional training (e.g., teacher education)—can override the need for other SEC skills. It also challenges the implicit assumption in the Big Five Personality Model (McCrae and John, 1992) that “extroversion” (linked to RS) or “conscientiousness” (linked to SM) are necessary for engagement, showing that internal self-awareness can be a viable alternative.

Collectively, the three solutions confirm, refine, and extend existing knowledge:

Solution 1 and solution 2 validate the “configurational logic” of SEC (no single dimension is necessary, and synergies drive outcomes) observed in prior fsQCA studies (Song et al., 2024; Ma et al., 2024) and theoretical frameworks (SDT, Broaden-and-Build Theory).

Solution 3 addresses a gap in the literature by identifying SA as a standalone core condition—an outcome not documented in prior SEC-participation research, and one that highlights the importance of group-specific traits (e.g., teacher education students’ reflective practice) in shaping SEC’s effects.

These findings reinforce that SEC’s impact on participation is not uniform but context-dependent, with different dimensions serving as “core enablers” based on the nature of engagement (effort-focused vs. social-focused vs. self-directed) and the characteristics of the student group.

### 6.2 Theoretical implications

Expanding the Configurational Mechanism of Social–Emotional Competence (SEC) this study enriches SEC theory by validating and extending its “equifinal” influence on classroom participation. Paths 1 (SM as core) and 2 (RS as core) confirm SEC dimensions typically function through synergies—aligning with Self-Determination Theory (SDT) and the Broaden-and-Build Theory. More critically, Path 3 (SA as the sole core) breaks new ground: it identifies SA as a standalone driver, challenging the prior assumption that SEC relies only on multi-dimensional interactions. This supplements the SEL framework (CASEL, 2023) by defining SA’s boundary conditions as an independent enabler (especially for goal-oriented groups like teacher education students) and broadens SEC’s theoretical scope beyond “synergy-only” models.

Refining the Theoretical Account of Student Engagement by integrating fsQCA with SDT and the Broaden-and-Build Theory, the study clarifies how SEC dimensions target distinct psychological/emotional mechanisms. For example, Path 1 links SM to SDT’s “competence” need (sustaining effort), while Path 3 ties SA to “autonomy” (driving self-directed participation)—nuances overlooked in traditional linear research (e.g., regression). Additionally, the irrelevance of certain SEC dimensions in specific paths (e.g., RDM in Path 1, SA in Path 2) highlights SEC’s context dependency, moving beyond one-size-fits-all engagement models to a more holistic, scenario-sensitive account.

Challenging Personality-Centric Engagement Frameworks the study questions the Big Five Personality Model’s (McCrae and John, 1992) implicit focus on fixed traits (extroversion linked to RS, conscientiousness linked to SM) as necessary for participation. Path 3 demonstrates that malleable SA can substitute for these traits, shifting theoretical focus from unchangeable personality to modifiable SEC skills—expanding the range of recognized engagement drivers and emphasizing SEC’s intervention potential.

### 6.3 Practical implications

Design of Targeted SEC Interventions Educators can develop tailored SEC training aligned with the three paths to address diverse student needs. For students struggling with sustained effort (e.g., distraction), prioritize SM training (e.g., time-management workshops) paired with SA/RS exercises to align effort with goals and social contexts. For socially oriented learners, center RS development (e.g., collaborative tasks) alongside SOA training to enhance adaptive interactions. For self-directed students (common in teacher education), design SA-focused activities (e.g., reflective journals on participation goals) to leverage intrinsic motivation, even if other SEC skills are weak.

Optimization of Teacher Education Curricula given the sample’s focus, teacher education programs can integrate SEC into professional training to support students’ current participation and future teaching practice. Embed SM modules in pedagogy courses to help future teachers model self-regulation for K-12 students; incorporate RS simulations (e.g., discussion facilitation) to build skills critical for both student engagement and teaching; and strengthen SA training via peer feedback and self-assessment tools—leveraging Path 3’s insight into SA’s unique value for this population’s growth.

Development of Inclusive Classroom Participation Strategies Instructors can adopt multi-path support to accommodate varied SEC profiles. Offer a mix of effort-focused (e.g., structured task participation) and social-focused (e.g., group debates) activities to match Paths 1 and 2. For students relying on Path 3, provide self-directed options (e.g., pre-submitted questions, individual reflections shared with the class) to enable comfortable engagement—ensuring all students can participate by leveraging their SEC strengths.

## 7 Conclusion

This study used fsQCA to explore the effects of social–emotional competence on classroom participation of university students, and explored the effects of each component of social–emotional competence on classroom participation. The study showed that social–emotional competence is the basic element of classroom participation of university students, and in three different solutions, self-determination, relationship skills, and self-awareness are the core conditions of classroom participation of university students, respectively. The theoretical significance of this study is for the researcher to understand classroom participation from the micro perspective of social–emotional competence. The practical value of the study is that it provides an important reference for teachers to better understand the influencing factors of university students’ classroom participation, and at the same time, it enables students to be more explicit about their social–emotional competence, and then pay attention to the improvement of their social–emotional competence.

## 8 Limitation and future research

### 8.1 Research limitations

Narrow focus on antecedent factors: This study only explored social–emotional competence (SEC) as the driver of classroom participation, ignoring the joint effects of environmental factors (e.g., class size, classroom climate) and context-individual interactions (e.g., how teacher assessment strategies moderate SEC’s impact). This restricts the comprehensiveness of causal mechanisms.

Limited sample generalizability: The sample was solely teacher education students, whose professional traits (e.g., reflective practice training) amplify self-awareness (SA)‘s role. Findings may not apply to non-education majors (e.g., science, business) with different academic demands and career orientations.

Cross-sectional design constraints: One-time questionnaire data fails to capture dynamic changes in SEC-participation configurations over time or rule out reverse causality (e.g., whether high participation enhances SEC), weakening causal inference.

### 8.2 Directions for future research

Incorporate multi-layered factors: Integrate environmental variables (e.g., classroom climate) and other individual traits (e.g., academic self-efficacy) into the fsQCA model to explore cross-level configurational effects (e.g., whether “high SA + supportive climate” forms a new participation path).

Conduct cross-disciplinary comparisons: Extend samples to students of different majors (e.g., engineering, humanities, business) to compare SEC-participation configurations. For example, test if engineering majors rely more on self-management (SM) for project-based learning, while humanities majors prioritize SA for critical reflection, clarifying discipline-specific boundary conditions.

Adopt longitudinal and mixed methods: Use longitudinal data (e.g., semester-long tracking) to capture dynamic SEC-participation links. Combine fsQCA with interviews to explain path differences (e.g., why SA alone drives participation in teacher education but not engineering), strengthening research rigor.

## Data Availability

Data are available from the corresponding author upon reasonable request.

## Footnotes

1
https://www.wjx.cn
